# Supplementary material for: A TRAF-like E3 ubiquitin ligase TrafE coordinates ESCRT and autophagy in endolysosomal damage response and cell-autonomous immunity to Mycobacterium marinum
Source: eLife. 2023 Apr 18;12:e85727. doi: 10.7554/eLife.85727 (PMC10181826; doi:10.7554/eLife.85727)
Supplement: Supplementary file 1. — (a) D. discoideum material used in this study. The Supplementary file includes table with the D. discoideum strains used in this study, the overexpression plasmids and the plasmids used for generation of trafE knock-out and GFP knock-in. (b) M. marinum material used in this study. The Supplementary file includes table with the M. marinum strains and M. marinum plasmids used in this study. (c) Primers used in this study. The Supplementary file includes table with primers used to amplify trafA, trafB, trafC and trafE CDSs, primers used for knock-out, GFP knock-in generation, act5 locus integration and screenings. [file elife-85727-supp1.docx]

| **Supplementary File 1 – supplementary file 1a. *D. discoideum* material used in this study.** | | |
| --- | --- | --- |
| **Strain** | **Characteristics** | **Source / Reference** |
| Ax2(Ka) | wt, parental strain of the *trafE* KO, *atg1* KO and GFP-trafE KI |  |
|  |  |  |
| **Plasmids** |  |  |
| GFP-TrafA | pDM317-trafA, G418^r^ | This study |
| GFP-TrafB | pDM317-trafB, G418^r^ | This study |
| GFP-TrafC | pDM317-trafC, G418^r^ | This study |
| GFP-TrafD | pDM317-trafD, G418^r^ | This study |
| GFP-TrafE | pDM317-trafE, G418^r^ | This study |
| dsRed-TrafE | pDM318-trafE, G418^r^ | This study |
| ALIX-GFP | pDM1515-ALIX, Hyg^r^ | This study |
| GFP-Vps32 | pDM1513-Vps32, Hyg^r^ | This study |
| GFP-Vps4 | pDM1513-Vps4, Hyg^r^ | This study |
| GFP-Atg8a | pDM1513-Atg8a, Hyg^r^ | This study |
| mCherry-Plin | pDM1042-plin, Hyg^r^ | This study |
| pKOSG-trafE_KO | *trafE* knock-out plasmid, Blasticidin^r^ | This study |
| pPI183-trafE | *trafE* GFP knock-in plasmid, G418^r^ | This study |

| **Supplementary File 1 – supplementary file 1b. *M. marinum* material used in this study.** | | |
| --- | --- | --- |
| **Strain** | **Characteristics** | **Source / Reference** |
| M | wt, parental strain | L. Ramakrishnan  (University of Cambridge) |
| ∆RD1 | RD1 locus ablation mutant | L. Ramakrishnan  (University of Cambridge) (Volkman et al., 2004) |
|  |  |  |
| **Plasmids** |  |  |
| pMSP12::DsRed/GFP | DsRed/GFP under control of the msp12 promoter, Kan^R^ | Addgene #30171 and #30167 (Cosma et al., 2004) |
| pCherry10 | mCherry under control of the G13 promoter, Hyg^r^ | Addgene #24664 (Carroll et al., 2010) |
| pMV306hsp+LuxG13 | Luciferase under control of the G13 promoter, Kan^R^ | Addgene # 26161 (Andreu et al., 2010) |

| **Supplementary File 1 – supplementary file 1c. Primers used in this study.** | | |
| --- | --- | --- |
| **Primers** | **Sequence (5’-3’)** | **Purpose** |
| LR7F | CAGGATCCAAAATGGATATTTCTCAAATCC | DDB_G0272454 / *trafA* 5' BamHI |
| LR7R | CAACTAGTATGTTTATCACATTGAGAC | DDB_G0272454 / *trafA* 3’ SpeI |
| LR8F | CAGGATCCAAAATGACAGAGTTTAAAATTAG | DDB_G0285149 / *trafB* 5' BamHI |
| LR8R | CAACTAGTTTTAGTAGTTAAAGGATC | DDB_G0285149 / *trafB* 3' SpeI |
| LR9F | CAGGATCCAAAATGTCAATTGATATAAAATTTAC | DDB_G0290883 / *trafC* 5' BamHI |
| LR9R | CAACTAGTAGACTCCAATGGTTCATATTC | DDB_G0290883 / *trafC* 3' SpeI |
| LR9/10F | CAGGATCCAAAATGTCAATTGATATAAAATTTAC | DDB_G0290883 / *trafD* 5' BamHI |
| LR10R | CAACTAGTAGACTCCAATGGTTCATATTC | DDB_G0290961 / *trafD* 3' SpeI |
| LR32F | CAGGATCCAAAATGACAGTAAAATATTCAATTAATG | DDB_G0290971 / *trafE* 5’ BamHI |
| LR32R | CAACTAGTTGGTAAAACTTGAATTCTAAG | DDB_G0290971 / *trafE* 3’ SpeI |
| LR60F | AGCGCGTCTCCAATG CTGCAG GAATTATTATCAATTTATTTAGGTTGGATTTGGTC | *trafE* KO left arm forward; CS1, PstI |
| LR60R | AGCGCGTCTCCGTTG GTTCATTAATTGAATATTTTACTGTCATTTTATG | *trafE* KO left arm reverse; CS2 |
| LR61F | AGCGCGTCTCCCTTC GTTATTGGTCTTAGAATTCAAGTTTTAC | *trafE* KO right arm forward; CS3 |
| LR61R | AGCGCGTCTCCTCCC CTGCAG CAATTTTATATTGGTGGTGTATTTGATATTC | *trafE* KO right arm reverse; CS4, PstI |
| LR62F | GTAATAATAATCAACAAGAACAAAAACAAGAATATC | *trafE* gDNA upstream of KO Left Arm |
| LR62R | CTGCTGATGCTGTTGAGTAAG | *trafE* gDNA downstream of KO Right Arm |
| LR63F | GAGTCTTGTAAAAAATCATTCCCAAG | *trafE* qPCR & wt-positive screen |
| LR63R | GTTGGTTATTTATAACTTTGTCCATC | *trafE* qPCR & wt-positive screen |
| LR68F | GAAGGTATTCAAATCCTAAAGGATAATATG | 3' of DDB_G0290973, upstream of *trafE*, use with BSR-R and qPCR primer LR63R |
| LR69F | CATGTGGTGTATCAAACTTTGTTTC | 3' of DDB_G0290957, downstream of *trafE*, use with BSR-F and qPCR primer LR63F |
| LR70F | CAAATGGTTGGTTAACTGATGAAG | wt-positive combine with LR62R |
| LR70R | GTAAAATTATCTGATAGTGATTCATGATCTAC | wt-positive combine with LR62F |
| LR71F | CAGGTACCGAGTCTTGTAAAAAATCATTCCCAAG | *trafE* 3' KpnI on 5' |
| LR71R | CAAGATCTTGGTAAAACTTGAATTCTAAGACCAATAAC | *trafE* 3' BglII on 3' |
| LR72F | CAGTCGACAATAAAAAAAAAAAATAAAAAAAACAAATCCAATTATTTTTATATTAAATAATTAAAATAAATGTAAC | *trafE* UTR 5' SalI |
| LR72R | CAGAGCTCCTGCTGATGCTGTTGAGTAAG | *trafE* UTR 3' SacI |
| LR99F | CAAGATCTATGGTTCATGTATCAAGCTTTAAAAAC | *Atg8a* 5' BamHI |
| LR99R | CTACTAGTTAAATCACTACCAAAAGTATTTTCACCAC | *Atg8a* 3' SpeI |
| LR105F | CA AGATCTAAA ATGAAACTCTTTGGTAAACCAAAACCAAAAC | *Vps32* 5' BamHI |
| LR105R | CTACTAGTCATTGCTAAAGATTCTTCCAAAGCTCTAATC | *Vps32* 3' SpeI |

Andreu, N., Zelmer, A., Fletcher, T., Elkington, P. T., Ward, T. H., Ripoll, J., Parish, T., Bancroft, G. J., Schaible, U., Robertson, B. D. & Wiles, S. 2010. Optimisation of bioluminescent reporters for use with mycobacteria. *PLoS One,* 5**,** e10777.

Carroll, P., Schreuder, L. J., Muwanguzi-Karugaba, J., Wiles, S., Robertson, B. D., Ripoll, J., Ward, T. H., Bancroft, G. J., Schaible, U. E. & Parish, T. 2010. Sensitive detection of gene expression in mycobacteria under replicating and non-replicating conditions using optimized far-red reporters. *PLoS One,* 5**,** e9823.

Cosma, C. L., Humbert, O. & Ramakrishnan, L. 2004. Superinfecting mycobacteria home to established tuberculous granulomas. *Nat Immunol,* 5**,** 828-35.

Volkman, H. E., Clay, H., Beery, D., Chang, J. C., Sherman, D. R. & Ramakrishnan, L. 2004. Tuberculous granuloma formation is enhanced by a mycobacterium virulence determinant. *PLoS Biol,* 2**,** e367.
